# Is it worth to perform initial non-operative treatment for patients with acute ACL injury?: a prospective cohort prognostic study

**DOI:** 10.1186/s43019-021-00094-3

**Published:** 2021-04-06

**Authors:** Yong-Geun Park, Chul-Won Ha, Yong-Beom Park, Sang-Eun Na, Manyoung Kim, Tae Seon Kim, Yong Yeon Chu

**Affiliations:** 1 Department of Orthopedic Surgery, Jeju National University Hospital, Jeju National University School of Medicine, Aran 13gil 15, Jeju-si, Jeju Self-Governing Province 63241 South Korea; 2 Department of Orthopedic Surgery, Samsung Medical Center, Sungkyunkwan University School of Medicine, 81 Irwon-ro, Gangnam-gu, Seoul, 06351 South Korea; 3 Stem Cell & Regenerative Medicine Institute, Samsung Medical Center, 81 Irwon-ro, Gangnam-gu, Seoul, 06351 South Korea; 4 Department of Health Sciences and Technology, SAIHST, Sungkyunkwan University, 81 Irwon-ro, Gangnam-gu, Seoul, 06351 South Korea; 5 Department of Orthopedic Surgery, Chung-Ang University Hospital, Chung-Ang University College of Medicine, 102 Heukseok-ro, Dongjak-gu, Seoul, 06973 South Korea; 6 Department of Orthopedic Surgery, Asan Teun Teun Hospital, 593 2-dong, Sangrok-gu, Ansan-si, Gyeonggi-do 15512 South Korea; 7 Department of Orthopedic Surgery, Wiltse Memorial Hospital, 560 Gyeongsu-daero, Dongan-gu, Anyang-si, Gyeonggi-do 14112 South Korea

**Keywords:** Non-operative treatment, Time of starting treatment, Acute injury, ACL, Knee

## Abstract

**Purpose:**

To evaluate the result of implementing an initial non-operative treatment program for an acute ACL injury and to find if the timing of initiating the non-operative treatment is significant.

**Methods:**

This study included a prospective cohort of 85 consecutive patients with acute ACL injury who were treated according to the above strategy for the initial 3 months with 1-year follow-up. Clinical evaluations were made by Lysholm score, Tegner activity score, Lachman test (LT), pivot-shit test (PST), and the side to side difference (SSD) by KT-2000 arthrometer. The results were analyzed according to the timing of initiating the non-operative treatment.

**Results:**

Initially, 84% of the patients showed LT and PST ≤ grade 1, and 16% with ≥grade 2. At 1-year follow-up, 77 patients (91%) with LT and PST ≤ grade 1 did not receive reconstruction as copers and 8 patients with LT or PST ≥ grade 2 required reconstruction (six patients received the operation and two refused). The patients with LT and PST ≤ grade 1 showed average Lysholm score 91.2, average SSD 2.5 mm, and mean Tegner score decreased from 6.9 (pre-injury) to 6.2. Patients who started the non-operative treatment within 2 weeks after injury revealed superior rates of grade 0 or 1 instability than those who commenced the treatment later than 2 weeks after injury (*P = 0.043*).

**Conclusions:**

Implementing a non-operative treatment with brace in acute phase of ACL injury appears to be an effective and viable option to achieve a reasonable clinical outcome. We recommend earlier initiation of the non-operative treatment to obtain a better result in patients with acute ACL injury.

**Supplementary Information:**

The online version contains supplementary material available at 10.1186/s43019-021-00094-3.

## Introduction

An anterior cruciate ligament (ACL) injury is one of the most common significant knee injuries during sports activities, however, the value of initial non-operative treatment for patients with acute ACL injury has not been properly recognized and is controversial [1–5]. ACL has primary healing potential and there have been reports of spontaneous healing of ACL after an injury, although the healing potential is less in complete ruptures [6]. Therefore, proper protection of the acutely injured ACL and ligament and prevention of potential impairments may result in a good result in patients with acute ACL injury in patients with acute ACL injury. Actually, there were reports of true copers after non-operative treatment even though it may not be a complete healing [7, 8].

The indication of non-operative treatment has been known to include patients with partial tears, young children with wide-open physes, and patients with low-risk activity levels, isolated ACL injuries, and mild pathologic laxity [9]. Non-operative management includes a short period of immobilization, bracing, a progressive rehabilitation program, and regular follow-up evaluations [1]. The various results of non-operative treatment of acute ACL injury previously reported may be due to the variability of important parameters. The previous reports are from different inclusion criteria and treatment durations, and interestingly, many of them actually did not specifically describe the definition in terms of the acuteness of the ACL injury or the timing of initiating the non-operative treatment [9–13]. Moreover, few studies analyzed both the ligamentous stability and functional outcome after non-operative treatment of acute ACL injury [14–18]. Furthermore, we could not find an article in the literature that compared the results from different timing of initiating the non-operative treatment after acute ACL injury.

Therefore, the purpose of this study was to evaluate the ligamentous stability as well as the functional status of patients after non-operative treatment of acute ACL injury, and to compare the outcome according to different timing of initiating the treatment; within or later than 2 weeks after the injury (to determine if the onset of treatment affects the outcome). We hypothesized that good ligament stability and functional status can be achieved through strict non-operative treatment program for an acute ACL injury; and the earlier the non-operative treatment is commenced, the better the clinical outcome can be obtained.

## Methods

**Fig. 1:**
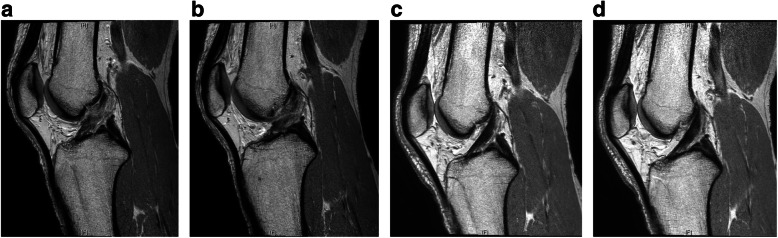
Non-operative treatment was performed on the 28 year-old male patient with an acute anterior collateral ligament (ACL) injury that had occurred while playing soccer 1 week ago. **a**, **b** T2-weighted magnetic resonance (MR) sagittal image of the knee indicates a midsubstance ACL rupture. Initial physical examination was Lachman test grade 1 and pivot-shift test grade 1, and the Tegner activity score before the injury was 7.0. **c**, **d** T2-weighted MR image of the same patient treated non-operatively according to our program shows a well-healed ACL at 1 year after injury. At 2 years after the injury, physical examination was Lachman test grade 0 and pivot-shift test grade 0, and the Tegner activity score was 7.0

**Fig. 2:**
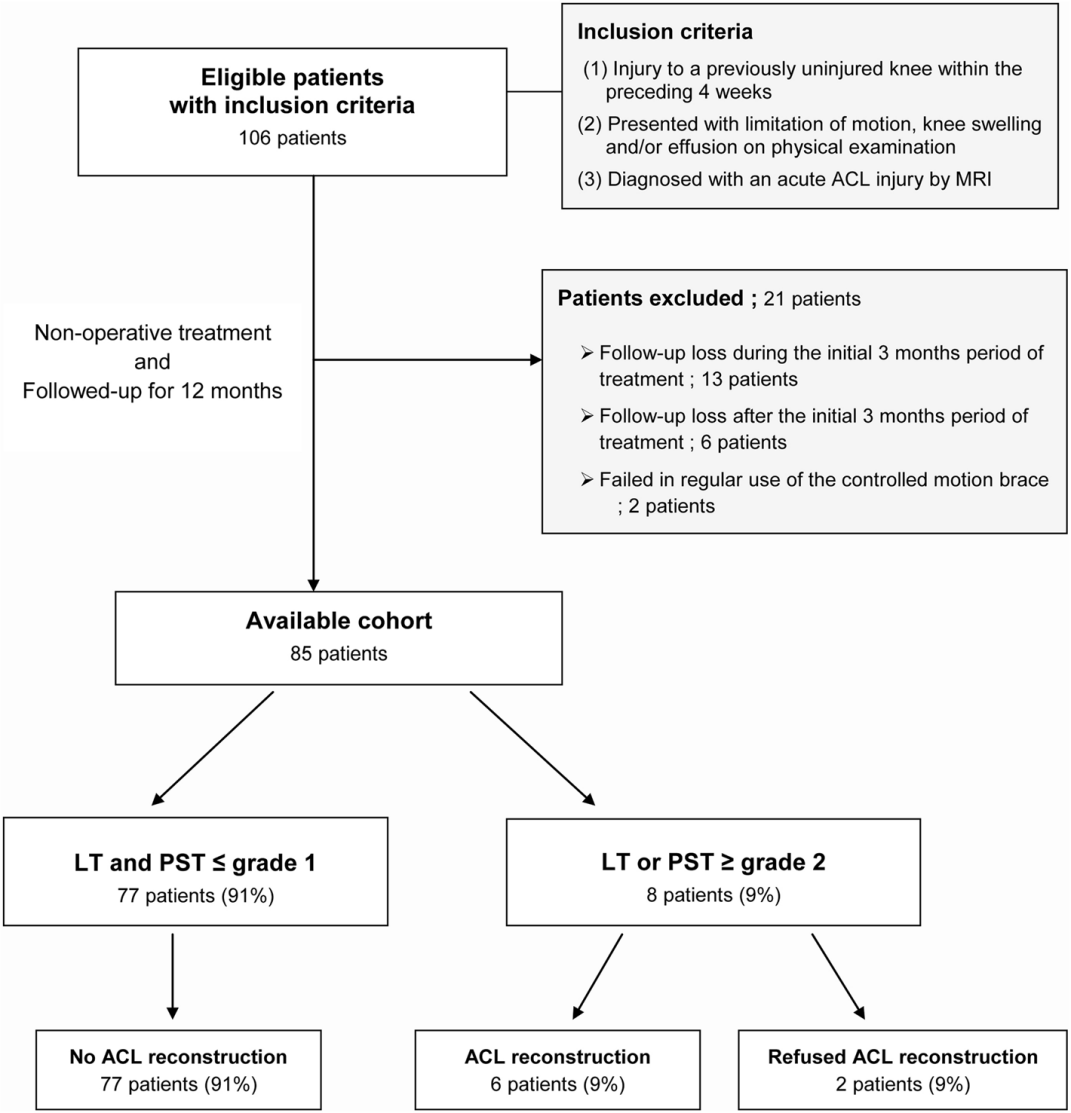
Flow of participants detailing inclusion at 1-year follow-up. *LT* Lachman test, *PST* pivot-shift test, *ACL* anterior cruciate ligament

Two hundred and ninety eight patients with acute or chronic ACL injury were treated at our institution between April 2008 and February 2013. Of these, the inclusion criteria for the initial non-operative treatment for “acute ACL injury” were the followings: patients (1) who had significant trauma to a previously uninjured knee within the preceding 4 weeks, (2) who presented with limitation of motion, swelling and/or effusion of the injured knee joint, and (3) whose diagnosis were confirmed as ACL injury by magnetic resonance imaging (MRI). Increased signal intensity and swelling of ACL substance on MRI was regarded as consistent with acute ACL injury [19] (Fig. 1). Patients who had previous history of a substantial injury to either the affected knee or the contralateral knee, or patients who required surgical treatment due to combined ligament or meniscus injuries were excluded. Consequently, the cohort of the present study initially consisted of 106 patients who met the inclusion criteria. The cohort was prospectively followed up at regular intervals for a minimum of 12 months. Among them, 85 (80%) patients were available for regular follow-up and 21 (20%) patients were excluded (Fig. 2).

Our non-operative treatment program consisted of 6 phases (Supplement 1). In phase I-III (from initial visit to 8 weeks), limited hinge brace was applied in order to prevent further injury, especially caused by pivot shifting motion, protect blood clot formation, and reduce anteriorly displacing force on tibia. Range of motion exercise, quadriceps setting and straight leg raising exercise were rigorously encouraged while wearing the brace. A gradual increase of exercise for improving muscle strength and endurance was implemented whilst maintaining adequate protection to allow healing of the injured ACL (Phase IV; until 12 weeks). The strength and sets of exercise were increased according to the capacity of each patient under the supervision of physical trainers. Upon successful completion of previous phases, the non-operative treatment protocol proceeds, involving active rehabilitation and exercises that ultimately prepare the patient to return to sports (Phase V-VI; until 12 months). At the end of phase IV (at 12 weeks into the protocol), the knee was examined with Lachman test (LT) and pivot-shift test (PST). If a hard endpoint was not palpable on LT or PST was positive of more than grade 2, surgical intervention was recommended. Among them, the patients who wanted conservative treatment rather than surgery were categorized as coper and they proceeded to phase V-VI. Home exercise was continued and rehabilitation counseling was done according to the knee stability and muscle strength at out-patient visits.

The clinical evaluation included LT, PST, Lysholm knee score, Tegner activity score and KT-2000 [20–22]. LT or PST was initially performed at the initial visit within 4 weeks of the injury, then at 3 months and at 1-year follow-up. The LT was graded as grade 0 (< 3 mm), grade 1 (3-5 mm), grade 2 (6-10 mm), and grade 3 (> 10 mm). The PST was graded as grade 0 (equal), grade 1 (glide), grade 2 (clunk), and grade 3 (marked) [23, 24]. The pre- and post-injury Tegner activity score was assessed at initial visit and at the 1-year follow-up. Lysholm knee score and side to side difference in anterior laxity by KT-2000 (MEDmetric, San Diego, CA) were also evaluated at 1-year follow-up.

Statistical analyses were performed with the SPSS for Windows 18.0 software package (SPSS Inc., Chicago, IL). Fisher’s exact test was utilized to evaluate the treatment results following the different timing of initiating the treatment.

## Results

The final cohort of this study comprised 85 patients (64 males and 21 females) with a mean age of 35.8 years (range 18–59). The average activity level prior to injury was Tegner activity score 7.0 (range 5–8). The most common cause of the injury was a sports injury; soccer (30 patients), non-sports slip (16), skiing (15), basketball (5), martial arts (4), running (4), traffic accident (2), badminton (2), tug-of-war (2), lacrosse (1), ice hockey (1), foot bolley ball (1), tennis (1) and falling off a ladder (1). There were 27 patients (31%) with isolated ACL injury and 43 patients (50%) with combined ligamentous injuries; medial collateral (36), posterior cruciate (2), lateral collateral (1), medial collateral + posterior cruciate (2), and medial + lateral collateral (2) ligaments. There were 31 patients (35%) who had combined meniscal injuries; medial meniscus (20), lateral meniscus (8) and medial + lateral meniscus (3) in the study cohort. Out of the 31 patients with coexisting meniscal tears, 21 were asymptomatic after the non-operative treatment of ACL, but 10 required arthroscopic meniscectomy or repair after the initial non-operative treatment to heal the ACL. Any patients who required acute surgical treatment (e.g. meniscal tears that require early surgery such as displaced bucket handle tears) were excluded from this study cohort (Patients belong to exclusion criteria).

**Fig. 3:**
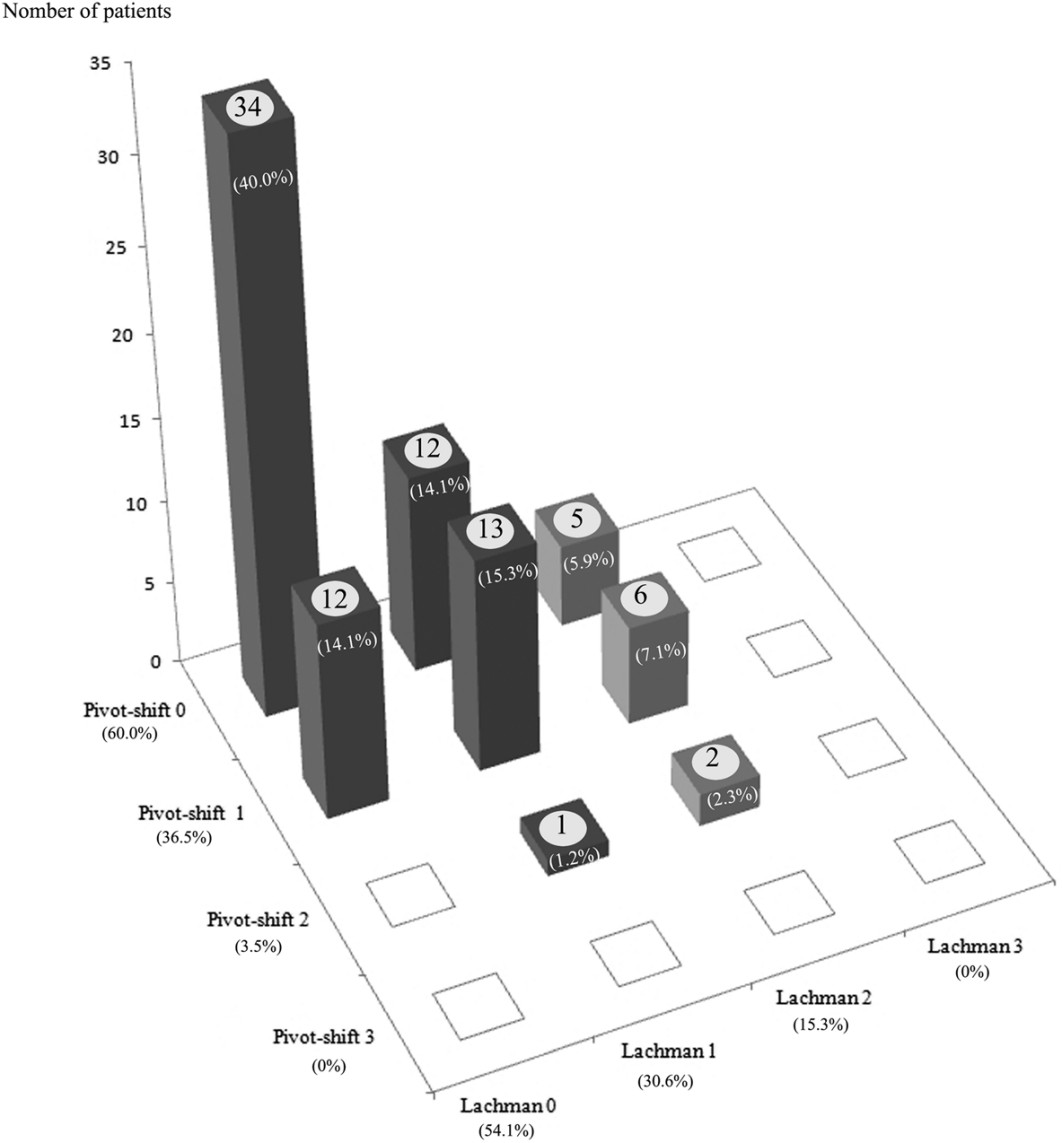
Distribution of patients according to Lachman test and pivot-shift test within 4 weeks after the anterior cruciate ligament (ACL) injury. Pivot-shift 0, pivot-shift test grade 0; Lachman 0, Lachman test grade 0

Initially, 84% of the patients showed LT and PST ≤ grade 1, and 16% with ≥grade 2 (Fig. 3). The initial LT was grade 0 in 46 patients (54%), which improved to grade 0 in 71 patients (84%) at 1-year follow-up. On PST, 51 patients (60%) showed grade 0 initially, which improved to 63 patients (74%) at 1-year follow-up. As a whole, 77 patients (91%) showed LT and PST ≤ grade 1 (53 patients with both the LT and PST grade 0) at 1 year follow-up (Table 1). Stability on physical examination was maintained or improved in 68 (80%) of the 85 patients in this study cohort. Of the 34 patients with both LT and PST grade 0 initially, the stability was maintained in 26 patients (76%) at 1-year follow-up. Of the 14 patients with initial LT or PST ≥ grade 2, 11 (79%) showed improved stability (Table 2).

**Table 1:** Comparison of the number of cases between the initial examination and at 1-year follow-up

	Lachman test		Pivot-shift test	
	Initial	At 1-year follow-up	Initial	At 1-year follow-up
Grade 0 (%)	46 (54%)	71 (84%)	51 (60%)	63 (74%)
Grade 1 (%)	26 (31%)	13 (15%)	31 (36%)	15 (17%)
Grade 2 (%)	13 (15%)	1 (1%)	3 (4%)	5 (6%)
Grade 3 (%)	0	0	0	3 (3%)
Total (%)	85 (100%)	85 (100%)	85 (100%)	85 (100%)

**Table 2:**
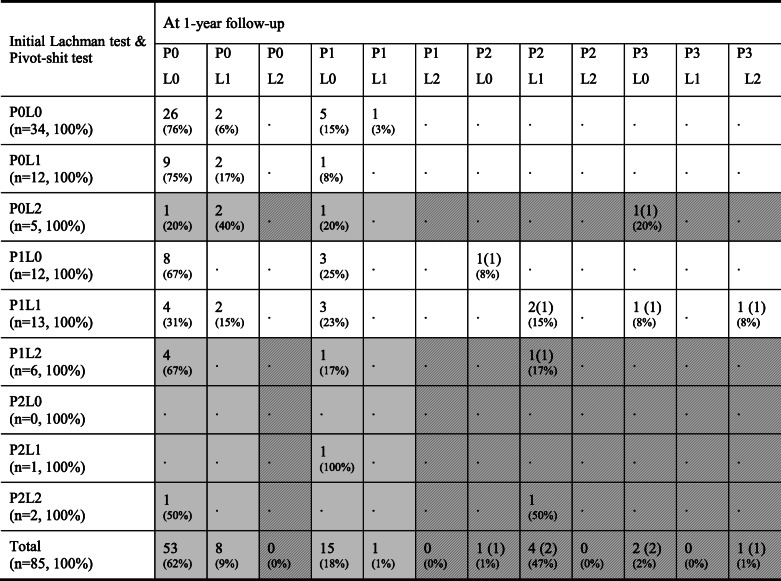
Results of the non-operative treatment according to the initial Lachman and pivot-shift tests

L* Lachman test grade *, P* pivot-shift test grade * (i.e., P0L0 = pivot-shift test grade 0 and Lachman test grade 0).

(_): number of patients who underwent an anterior cruciate ligament (ACL) reconstruction described in parentheses. Patients undergoing ACL reconstruction were examined prior to the surgery.

: Patients initially with grade ≥ 2 instability on either Lachman test or pivot-shift test improved to grade ≤ 1 instability (both Lachman and pivot-shift test) at 1-year follow-up with non-operative treatment.

: Patients initially grade ≥ 2 instability on either Lachman test or pivot-shift test remained at grade ≥ 2 instability (either Lachman or pivot-shift test) at 1-year follow-up with non-operative treatment.

At 1-year follow-up, 77 (91%) out of the 85 patients after the non-operative treatment did not receive reconstruction as copers (patients with LT and PST ≤ grade 1) and 8 (9%) patients required reconstruction (patients with LT or PST ≥ grade 2). Eight patients were recommended to undergo an ACL reconstruction. Although 6 of the 8 patients underwent the ACL reconstruction, the remaining two patients, who had no complaint of functional instability in their daily activities, refused the surgery probably due to their low activity demand or adaptation with activity modification (Fig. 2).

After the initial phase 1 treatment with a brace for 8 weeks, 29 patients did not demonstrate a hard end point on LT. They were advised to wear the brace for an additional 4 weeks. After the additional 4 weeks of bracing, 12 showed improvement in stability, 15 indicated no change, and 2 demonstrated worsened stability.

Seventy-seven patients with LT and PST ≤ grade 1 showed Lysholm score of average 91.2 (range, 68–100), the average side to side difference of 2.5 mm (range, 0.0–4.5), and the mean Tegner activity score was decreased from 6.9 (range, 4–9) before the injury to 6.2 at 1-year follow-up (range, 4–8) (Table 3).

**Table 3:** Tegner activity score, Lysholm knee score, and side-to-side difference in anterior laxity by the KT-2000 arthrometer in patients with both Lachman test and pivot-shift test ≤ grade 1

At 1-year follow-up	P0L0 (*n* = 53, 100%)	P0L1 (*n* = 6, 100%)	P1L0 (*n* = 17, 100%)	P1L1 (*n* = 1, 100%)	Total (*n* = 77, 100%)
Changes of the Tegner activity score					
Improved	0	0	0	0	0
Maintained	27 (51%)	5 (83%)	7 (42%)	0	39 (51%)
Decreased (− 1 level)	17 (32%)	1 (17%)	5 (29%)	1 (100%)	23 (30%)
Decreased (− 2 level)	8 (15%)	0	5 (29%)	0	14 (18%)
Decreased (− 3 level)	1 (2%)	0	0	0	1 (1%)
Tegner activity score					
Pre-injury	6.7 (range, 4–8)	7.3 (range, 7–8)	7.3 (range, 5–9)	8.0	6.9 (range, 4–9)
Last follow-up	6.0 (range, 4–8)	7.2 (range, 7–8)	6.4 (range, 5–8)	7.0	6.2 (range, 4–8)
Lysholm knee score	91.2 (range, 72–100)	91.2 (range, 85–100)	91.4 (range, 68–100)	85	91.2 (range, 68–100)
Side-to-side difference (mm)	2.2 (range, 0–4.5)	2.8 (range, 1–4.5)	3.2 (range, 1.5–4.5)	4.5	2.5 (range, 0–4.5)

L* Lachman test grade *, P* pivot-shift test grade * (i.e., P0L0 = pivot-shift test grade 0 and Lachman test grade 0)

The comparison analysis of patients who initiated the non-operative treatment within or later than 2 weeks after the ACL injury revealed that there were more chance of resulting in both the LT and PST ≥ grade 2 in patients with delayed initiation of the non-operative treatment (*P* = 0.043) (Table 4).

**Table 4:** Distribution of patients with both Lachman and pivot-shift test ≤ grade 1 according to the time of initiating the non-operative treatment

Initial Lachman test and pivot-shift test	Lachman and pivot-shift ≤ G1(at 1-year follow-up) according to the time of initiating the treatment				
	< 1 week	1 ~ < 2 weeks	2 ~ < 3 weeks	3 ~ < 4 weeks	
L0P0 (*n* = 34)	11/11 (100%)	10/10 (100%)	4/4 (100%)	9/9 (100%)	
L0P1 (*n* = 12)	2/2 (100%)	0/0	3/3 (100%)	6/7 (85%)	
L1P0 (*n* = 12)	4/4 (100%)	5/5 (100%)	1/1 (100%)	2/2 (100%)	
L1P1 (*n* = 13)	3/4 (75%)	3/3 (100%)	1/2 (50%)	2/4 (50%)	
L0P2 (*n* = 0)	0/0	0/0	0/0	0/0	
L2P0 (*n* = 5)	4/4 (100%)	0/0	0/0	0/1 (0%)	
L1P2 (*n* = 1)	1/1 (100%)	0/0	0/0	0/0	
L2P1 (*n* = 6)	4/4 (100%)	0/0	0/0	1/2 (50%)	
L2P2 (*n* = 2)	1/1 (100%)	0/0	0/1 (0%)	0/0	
Total (*n* = 85)	30/31 (96.8%)	18/18 (100%)	9/11 (81.8%)	20/25 (80.0%)	*P* = 0.043

L* Lachman test grade*, P* pivot-shift test grade* (i.e., P0L0 = pivot-shift test grade 0 and Lachman test grade 0)

## Discussion

The results of this study shows that initial non-operative treatment of acute ACL injury implemented in acute phase (within 4 weeks after the injury) of ACL injury can result in a reasonable success. Non-operative treatment using a controlled motion brace initiated within 4 weeks of the injury showed 91% of both LT and PST ≤ grade 1 (62% with both LT and PST grade 0). Among them, decrease in Tegner activity score was less than 1. Only 9% (8 patients) out of the 85 patients resulted in more than grade 1 residual instability after the non-operative treatment, for whom we consequently recommended ACL reconstruction. Another important finding from the current study is that patients who initiated the non-operative treatment within 2 weeks after the ACL injury showed better chance of resulting in both the LT and PST ≤ grade 1. The high success rate of non-operative treatment in this study may be attributed to the considerable proportion of patients with LT0 and PS0 (62%). Due to swelling and effusion, instability could not be confirmed at initial visit. Initial evaluation for stability was done within 4 weeks allowing time for swelling and effusion to subside. For patients who showed LT0/PS0 within 4 weeks, non-operative treatment protocol was continued. We suspect that a sizable proportion of patients with LT0 and PS0 probably had partial ruptures rather than complete ruptures.

The natural history of ligament healing is similar to other vascular soft tissues, occurring in three identifiable stages: haemorrhage and inflammation, proliferation and scar formation, and remodeling. Each of the stages of healing depends upon an adequate blood supply for the delivery and removal of cells and metabolic substrates at the site of injury [25]. A number of studies found that the epiligament is a donor of fibroblasts and other connective tissue cells, progenitor cells and blood vessels, which migrate towards the body of the ligament via the endoligament and play a key role in the process of ligament repair [26]. Non-operative treatment initially performed by immobilization and protection using a brace is to avoid external factors that interfere with this healing process. It was followed by a rehabilitation program to recover joint motion, muscle strengthening, and activity level. There is no evidence yet whether the non-operative treatment affected the healing process, and the protocols used in previous studies have been tried in various ways without a specific guideline [1].

There are some previous papers that reported satisfactory results of an injured ACL using non-operative treatment. Ageberg et al. [14] and also Nueman et al. [13] reported that a well-constructed rehabilitation program with non-operative treatment resulted in satisfactory clinical outcomes after 15 years of follow-up. Ageberg et al. [14] also reported that 65 ~ 85% of 100 patients treated non-operatively demonstrated limb symmetry index of higher than 90%. However, both studies had the inclusion criteria of within 5 days of the ACL injury, which seem to represent a too limited percentage of patients in a real clinical setting. They also lacked the evaluation on restoration of the stability of ACL directly by KT-2000, LT or PST. Unlike these two studies, our protocol used a controlled motion brace during the treatment period, providing proper protection to the injured ACL. We agree that injured ACL has healing potential as mentioned by these studies, and we suggest that use of controlled motion brace could expand non-operative treatment to patients within 4 weeks after the injury. The result of the present study shows that a proper protection initiated within 4 weeks of ACL injury followed by careful rehabilitation can result in a good clinical outcome.

Since it is difficult to differentiate partial versus complete tears by MRI or physical examination at acute phase of ACL injury, we think that treating the two groups differently at acute stage of ACL injury is not possible in an actual clinical setting [9, 27, 28]. Neusel et al. [18] reported that good to satisfactory results were shown in cases of partial rupture, but the results were limited to isolated ACL injuries. Ahn et al. [10] reported a short term result of acute ACL injury after non-operative treatment using a controlled motion brace that showed improvement of ligament laxity and restoration of their continuity on MRI. However, they did not specifically describe the inclusion criteria of their non-operative treatment in terms of the “acuteness” of the ACL injury as well as the timing of initiating the non-operative treatment. Moreover, they just included patients with mild instability (LT 0 or 1) at initial visits and excluded patients with MRI findings suggesting complete rupture of ACL. Considering the limited accuracy in distinguishing whether the ACL injury is partial or complete by MRI in acute stage of ACL injury, and the difficulty in precisely assessing the stability at the acute stage of ACL injury [9, 27, 28], we think that their study seems to have a significant limitation. As we included every consecutive patient in a non-selective manner, we believe the strategy and result of the present study is more practical and informative. By the way, even in the cohort of this study, majority of patients showed mild instability initially, we admit that most of them should have had partial tear of the ACL.

In the present study, patients who initiated the non-operative treatment within 2 weeks after the ACL injury showed better chance of resulting in both the LT and PST ≤ grade 1. This result shows that the time of initiating the non-operative treatment is an important factor affecting the clinical outcome. Because previous reports on the non-operative treatment did not specify when the non-operative treatment was commenced, we believe the result of this study is significant by suggesting the guideline in terms of the timing of initiation of the non-operative treatment. We could consider initiating the non-operative treatment as soon as possible based on the result of this study.

The result of this study provides a rough prognosis of treatment according to the initial status of the knee after the acute ACL injury. Patients who initially showed both the LT and PST grade 0 had higher propensity to show LT and PST ≤ grade 1 after the non-operative treatment than the patients with initial LT or PST ≥ grade 2. However, even among the 14 patients with initial LT or PST ≥ grade 2, 11patients (79%) showed improved results with LT and PST ≤ grade 1. This finding suggests that there could have been a remodeling of the injured ACL along with the healing of the injured site. Kostogiannis et al. [16] reported that a positive PST at 3 months after injury is the strongest predictor for the need of reconstruction. We agree with their conclusion and we believe that the need of an ACL reconstruction should be determined after proper non-operative treatment in acute stage of ACL injury. In a randomized controlled trial, Frobell et al. [2] demonstrated that a strategy of rehabilitation plus early ACL reconstruction did not result in better outcome than a strategy of rehabilitation plus optional delayed ACL reconstruction after 2 years of follow-up. They emphasized that more than half the ACL reconstruction could be avoided without adversely affecting outcomes when the latter strategy is utilized. We believe the result of the present study supports their conclusion. Moreover, many of the patients in present study were able to return to their pre-injury activity level or had a slight decreased activity, which is consistent with the reports of Kostogiannis et al. [16] and Neuman et al. [17]. Therefore, we believe that in most patients with leisure sports activities, determination of the necessity of surgical reconstruction of ACL would better be made after completion of initial non-operative treatment in acute stage of ACL injury.

Some limitations of our study need to be addressed. First, we need to address that the initial instability of the study cohort was biased to low or moderate grade in LT and PST. There were relatively small numbers of patients (17% of the study cohort) with instability of more than LT or PST grade 1. Since patients with instability of less than LT or PST grade 2 would likely have been partial tears, we assumed that the proportion of partial tear was high in this study cohort. Second, we cannot guarantee that the long term clinical outcome of the patients with grade 1 instability without surgery will be well. However, Neuman et al. [17] reported that a low grade ACL injury appears to result in a successful outcome without secondary knee surgery or knee osteoarthritis in 15 years. Third, 21 patients (20%) out of the initial cohort of 106 patients were lost to follow-up. Thirteen patients were lost in follow-up during the initial three months period of the non-operative treatment, 2 patients failed in the regular use of a controlled motion brace, and 6 patients were gone missing in follow-up after the initial 3 month non-operative treatment period (Fig. 2). For this study, we made great efforts to contact the 21 patients lost to follow-up, which found that 5 underwent ACL reconstruction surgery at other hospital before completion of our initial non-operative treatment program, another 11 did not show any symptoms of instability, and 5 were unreachable. Fourth, we presented the instability degree using KT-2000 as an objective test for evaluating the recovery of injured ligaments. The lack of more objective results was a limitation of this study because we did not present followed-MRI results that can be displayed as images.

## Conclusion

Implementing a non-operative treatment with brace in acute phase of ACL injury appears to be an effective and viable option to achieve a reasonable clinical outcome. We recommend earlier initiation of the non-operative treatment to obtain a better result in patients with acute ACL injury.

## Supplementary Information

**Additional file 1: Supplement 1.** Non-operative treatment protocol for acute ACL injury in SMC (Samsung Medical Center) Knee Clinic (version 1.0.)

## Data Availability

The datasets generated and/or analyzed during the current study are not publicly available due to protection patients` information but are available from the corresponding author on reasonable request.

## Abbreviations

ACL: Anterior cruciate ligament
MRI: Magnetic resonance imaging
LT: Lachman test
L*: Lachman test grade *
PST: Pivot-shit test
P*: Pivot-shift test grade *
SSD: Side to side difference
