# Supplementary material for: Is it worth to perform initial non-operative treatment for patients with acute ACL injury?: a prospective cohort prognostic study
Source: Knee Surg Relat Res. 2021 Apr 6;33:11. doi: 10.1186/s43019-021-00094-3 (PMC8025569; doi:10.1186/s43019-021-00094-3)
Supplement: Supplementary file 1 — Additional file 1: Supplement 1. Non-operative treatment protocol for acute ACL injury in SMC (Samsung Medical Center) Knee Clinic (version 1.0.) [file 43019_2021_94_MOESM1_ESM.pdf]

SUPPLEMENT 1. Non-operative treatment protocol for acute ACL injury in SMC (Samsung Medical Center) Knee Clinic (version 1.0.)

**Phase (weeks)**	**I (-2 weeks)**<br>Immediate Intervention	**II (2-6 weeks)**<br>Early Intervention	**III (6-8 weeks)**<br>Late Intervention	**IV (8-12 weeks)**<br>Transitional Phase		**V (3-6 months)**<br>Preparation Phase	**VI (6-12 months)**<br>Return to Sports
				(8-10 weeks)	(10-12 weeks)		
1. Management of pain and swelling	- Cryotherapy<br>- Compression and elevation	- Cryotherapy	- Cryotherapy				
2. Protection	- Brace 0-90<br>- Crutches<br>- Wt bearing ambulation as tolerable	- Brace application<br>(~ 4 weeks: 0-120,<br>~ 6 weeks: 0-135)<br>- Crutches:<br>(*cease during 2~4 weeks)<br>- Full wt bearing ambulation	- Brace application<br>(~ 8 weeks: 0-135)<br>- No Crutches<br>- Full wt bearing ambulation	- Brace, (as required)<br>:**Instability(+) → apply<br>Instability(-) → no brace<br>- Full wt bearing ambulation	- Brace, (as required)<br>: Instability(+) → apply<br>Instability(-) → no brace<br>- Full wt bearing ambulation		
3. ROM and stretching	- ROM full extension<br>- ROM 0-90<br>- Patellar mobilization<br>- Stretching calf and hamstring	- ROM full extension<br>- PROM and AAROM<br>- Patellar mobilization<br>- Stretching calf and hamstring	- ROM full extension<br>- PROM and AAROM<br>- Full ROM<br>- Stretching calf and hamstring	- ROM full extension:<br>- AAROM<br>- Full ROM<br>- Stretching calf and hamstring	- ROM full extension:<br>- AAROM<br>- Full ROM<br>- Stretching calf and hamstring	- ROM full extension:<br>- Full ROM<br>- Stretching required	- ROM full extension:<br>- Full ROM<br>- Stretching required
4. Muscle strengthening exercise	- Q set and SLR<br>(brace applied state)	- Q set and SLR<br>(brace applied state 15-75)<br>- Heel-raise/toe-raise<br>- Isometric training (60,30)<br>- OKC exercise<br>1) Active knee flexion<br>(hamstring curls)<br>light resistance 10-70	-Resistance SLR as tolerable<br>(ankle wt. < 10% of body wt.)<br>- Heel-raise/toe-raise<br>- Isometric training<br>(90,60, 30)<br>- OKC exercise<br>1) Active knee extension<br>2) Active knee flexion	- SLR with rubber tubing<br>- Isometric training<br>(90, 60, 30)<br>- OKC exercise<br>1) Active knee extension<br>with resistance, 90-30<br>2) Active knee flexion<br>(hamstring curls)<br>with resistance 0-90<br>- Progressive, resistance to weight machine as tolerated<br>- †Aerobic conditioning	- SLR with rubber tubing<br>- Isometric training<br>(90, 60, 30)<br>- CKC exercise<br>1) Mini-squats 10-70<br>2) Wall slides 10-70<br>3) Leg press 10 –70<br>- OKC exercise<br>1) Active knee extension 9-0<br>2) Active knee flexion<br>Progressive, resistance to weight machine as tolerated<br>- Aerobic conditioning	- Muscle strengthening advancement<br>(OKC/CKC)<br>1) Bilateral to unilateral (CKC)<br>2) Increasing resistance<br>3) Increasing to full arc<br>-Aerobic conditioning	- Muscle strengthening<br>- Aerobic conditioning
5. Neuromuscular training		-Weight shifting exercise	- Balance training<br>1) Balance board<br>(two-legged)<br>2) Perturbation<br>(two-legged)	- Balance training<br>1) Balance board (two-legs)<br>2) Perturbation (two-legs)<br>3) Resistance band walking<br>4) Plyoback ball toss<br>5) Lateral step-up	- Balance training<br>1) Balance board (single-leg)<br>2) Perturbation (single-leg)<br>3) Resistance band walking<br>4) Plyoback ball toss<br>5) Lateral step-up	- Balance training<br>- Perturbation training	- Advance to balance and perturbation training (training depending on quad index)<br>- Advance to single-leg activities<br>eg.) balance with secondary activity
6. Tech to increase muscular strength, power and endurance					- Fast walk, Backward walk (straight, no jumping or cutting)<br>- Stair stepper/NordicTrack<br>- Bicycle exercise	- In-line jogging<br>(no jumping or cutting)<br>* progression of speed<br>: 1/2~full, backward run	- Start agility exercises<br>: gradually progress to sprinting and cutting drills<br>(training depending on quad index)

7. Functional exercise						- Functional training<br>1) Carioca<br>2) Lunges with sport cord<br>3) Forward/ backward running<br>4) In-place jogging w/ sports cord<br>5)Pool running	- Functional training<br>1)More challenging and emphasize correct movement patterns<br>2) Progress to sport specific plyometric training drills<br>→hop variations, skipping and bounding variations<br>→return to throwing program in overhead throwers, etc
8. Plyometrics					- Low level plyometrics<br>1) step and land<br>2) bilateral low level box drops	- Plyometric training<br>: box hops (double-leg)	- continue plyometric training
9. Agility and Sports specific exercises					- Initiate sport-specific exercise<br>1) cone drills<br>2) side shuffles<br>3) cariocas<br>- Walking-based agility exercises<br>- Low impact aerobic training<br>- Ergometer, elliptical, treadmill	- Basic agility exercises	- Gradual return to practice, starting with unopposed drills, progressing to opposed drills followed by return to competition<br>- [‡] Sports-specific drills
10. Prevention of recurrent injury							- Encourage maintenance program for strength/endurance
11. Milestones for progression to the next phase	A. Adequate wound healing<br>B. Controlled pain, swelling, effusion<br>C. Full passive extension 90 passive flexion<br>D. Good patellar mobility	A. Minimal pain and swelling<br>B. Full active extension w/o lag<br>C. Walking with full wt bearing, w/o crutches<br>D. Single stance on affected leg with upper extremity assisted<br>E. Isometric quadriceps strength at least 60% of contralateral knee (60,30)<br>E. [§]Clinical scoring	A. Normal gait<br>B. Isometric quadriceps strength at least 65% of contralateral knee (90, 60, 30)<br>C.Knee flexor/extensor strength ratio > 70-75 %<br>D. Hamstring strength equal	A.Performs activities of daily living<br>B.Increase strength and endurance	A. Adequate progression in neuromuscular balance training exercises<br>B. Tolerance for low impact aerobic activities<br>C. Single leg perturbation equivalent to contralateral leg<br>D. Isometric quadriceps strength (90, 60, 30)<br>: >70% of contralateral knee<br>E. Hamstring strength<br>: > 70% contralateral extremity<br>F. Single-leg function tests, hop distance, timed hop,<br>: >70% of contralateral knee<br>G. Clinical scoring	A. Ability to run for up to 1km without any pain or swelling or gait asymmetries<br>B. No subjective sense of instability<br>C. Isometric quadriceps strength (90, 60, 30), Single-leg function tests hop distance, timed hop<br>: >80% of contralateral<br>D. Isokinetic test (isometric + torque 300degree /sec, % difference) : 20~25%<br>E. Hamstrings/quadriceps ratio : > 70%<br>F. Joint arthrometer : <3mm<br>G. Clinical scoring	A. [§§]Criteria for return to sport<br>B. Clinical scoring

* Crutches Cease Criteria : 1) minimal pain and swelling, 2) full passive extension, 3) at least 100° to 120° flexion, 4) SLR without extension lag, 5) normal gait pattern without crutches
** Instability (+) : If a hard endpoint was not palpable on the Lachman test or pivot-shit test was positive of more than grade 2
† Aerobic conditioning : 1) Water walking 2) Stationary bicycling 3) Swimming 4) Ski machine
‡ Sports-specific drills : 1) Cone drills, 2) Side shuffles, 3) Grapevine drill, 4) Cariocas, 5) Sudden start/stops, 6) Agility drills combination, 7) Sport- and position- specific skill training
§ Clinical scoring : 1) SMC patellofemoral, 2) Tegner activity, 3) Lysholm, 4) Knee injury and osteoarthritis outcome score (KOOS)
§§ Criteria for return to sport: 1) Passive and active extension symmetrical to the noninvolved knee, 2) Flexion within 5 degrees of the noninvolved knee, 3) Minimal pain, no increase swelling with exercise, 4) Quadriceps index and Hop test greater than or equal to 90% of the contralateral knee, 5) Tolerating full effort agility, jumping, hopping, cutting, and sprinting activities without asymmetries or symptoms, 6) Full participation in practice

AAROM; active assistive range of motion, CKC; closed kinetic chain, OKC; open kinetic chain, PROM; passive range of motion, Q set; quadriceps setting exercise, ROM; range of motion, SLR; straight leg raise exercise, Wt; weight,
